# Prevention and treatment of oral adverse effects of antiresorptive medications for osteoporosis – A position paper of the Brazilian Society of Endocrinology and Metabolism (SBEM), Brazilian Society of Stomatology and Oral Pathology (Sobep), and Brazilian Association for Bone Evaluation and Osteometabolism (Abrasso)

**DOI:** 10.20945/2359-3997000000301

**Published:** 2020-10-21

**Authors:** Miguel Madeira, André Caroli Rocha, Carolina Aguiar Moreira, Águida Maria Menezes Aguiar, Sergio Setsuo Maeda, Abel Silveira Cardoso, Charlles Heldan de Moura Castro, Catarina Brasil D'Alva, Barbara Campolina Carvalho Silva, Bruno Ferraz-de-Souza, Marise Lazaretti-Castro, Francisco Bandeira, Sandra R. Torres

**Affiliations:** 1 Universidade Federal Hospital Universitário Clementino Fraga Filho Departamento de Clínica Médica Rio de Janeiro RJ Brasil Divisão de Endocrinologia, Departamento de Clínica Médica, Hospital Universitário Clementino Fraga Filho, Universidade Federal do Rio de Janeiro, Rio de Janeiro, RJ, Brasil; 2 Hospital Federal de Bonsucesso Departamento de Clínica Médica Divisão de Endocrinologia Rio de Janeiro RJ Brasil Divisão de Endocrinologia, Departamento de Clínica Médica, Hospital Federal de Bonsucesso, Rio de Janeiro, RJ, Brasil; 3 Universidade de São Paulo Faculdade de Medicina Hospital das Clínicas São Paulo SP Brasil Hospital das Clínicas, Faculdade de Medicina da Universidade de São Paulo, São Paulo, SP, Brasil; 4 Universidade Federal do Paraná Unidade de Endocrinologia e Metabologia (SEMPR) Curitiba PR Brasil Unidade de Endocrinologia e Metabologia (SEMPR), Universidade Federal do Paraná, Curitiba, PR, Brasil; 5 Secretaria Municipal de Saúde do Rio de Janeiro Hospital Municipal Souza Aguiar Rio de Janeiro RJ Brasil Hospital Municipal Souza Aguiar, Secretaria Municipal de Saúde do Rio de Janeiro, Rio de Janeiro, RJ, Brasil; 6 Universidade Federal de São Paulo Escola Paulista de Medicina Disciplina de Endocrinologia São Paulo SP Brasil Disciplina de Endocrinologia, Escola Paulista de Medicina, Universidade Federal de São Paulo, São Paulo, SP, Brasil; 7 Universidade Federal do Rio de Janeiro Faculdade de Odontologia Departamento de Patologia e Diagnóstico Oral Rio de Janeiro RJ Brasil Departamento de Patologia e Diagnóstico Oral, Faculdade de Odontologia, Universidade Federal do Rio de Janeiro, Rio de Janeiro, RJ, Brasil; 8 Universidade Federal de São Paulo Escola Paulista de Medicina Divisão de Reumatologia São Paulo SP Brasil Divisão de Reumatologia, Escola Paulista de Medicina, Universidade Federal de São Paulo, São Paulo, SP, Brasil; 9 Universidade Federal do Ceará Departamento de Medicina Clínica Fortaleza CE Brasil Departamento de Medicina Clínica, Universidade Federal do Ceará, Fortaleza, CE, Brasil; 10 Centro Universitário de Belo Horizonte Faculdade de Medicina Belo Horizonte MG Brasil Faculdade de Medicina, Centro Universitário de Belo Horizonte, Belo Horizonte, MG, Brasil; 11 Hospital Felício Rocho Divisão de Endocrinologia Belo Horizonte MG Brasil Divisão de Endocrinologia, Hospital Felício Rocho, Belo Horizonte, MG, Brasil; 12 Santa Casa de Belo Horizonte Divisão de Endocrinologia Belo Horizonte MG Brasil Divisão de Endocrinologia, Santa Casa de Belo Horizonte, Belo Horizonte, MG, Brasil; 13 Universidade de São Paulo Faculdade de Medicina Hospital das Clínicas São Paulo SP Brasil Laboratório de Endocrinologia Celular e Molecular (LIM-25) e Unidade de Doenças Osteometabólicas, Divisão de Endocrinologia, Hospital das Clínicas, Faculdade de Medicina, Universidade de São Paulo, São Paulo, SP, Brasil; 14 Universidade de Pernambuco Faculdade de Medicina Divisão de Endocrinologia e Diabetes Recife PE Brasil Divisão de Endocrinologia e Diabetes, Faculdade de Medicina da Universidade de Pernambuco, Recife, PE, Brasil

**Keywords:** Osteoporosis, bisphosphonate, medication-related osteonecrosis of the jaw, antifracture therapy, dental care

## Abstract

Antiresorptive therapy is the main form of prevention of osteoporotic or fragility fractures. Medication-related osteonecrosis of the jaw (MRONJ) is a relatively rare but severe adverse reaction to antiresorptive and antiangiogenic drugs. Physicians and dentists caring for patients taking these drugs and requiring invasive procedures face a difficult decision because of the potential risk of MRONJ. The aim of this study was to discuss the risk factors for the development of MRONJ and prevention of this complication in patients with osteoporosis taking antiresorptive drugs and requiring invasive dental treatment. For this goal, a task force with representatives from three professional associations was appointed to review the pertinent literature and discuss systemic and local risk factors, prevention of MRONJ in patients with osteoporosis, and management of established MRONJ. Although scarce evidence links the use of antiresorptive agents in the context of osteoporosis to the development of MRONJ, these agents are considered a risk factor for this complication. Despite the rare reports of MRONJ in patients with osteoporosis, the severity of symptoms and impact of MRONJ in the patients' quality of life make it imperative for health care professionals to consider this complication when planning invasive dental procedures.

## INTRODUCTION

The management of patients with osteoporosis taking antiresorptive drugs is challenging. While antiresorptive drugs are effective and well established in preventing osteoporotic fractures, dentists face difficult decisions when patients taking antiresorptive drugs require invasive dental treatment, mainly due to the risk of medication-related osteonecrosis of the jaw (MRONJ), a rare but severe adverse reaction associated with antiresorptive and antiangiogenic agents ( 1 – 4 ). Interprofessional collaboration is paramount in establishing strategies for improving clinical practice and better assisting these patients. Therefore, a need has arisen for a position paper involving health care professionals who assist patients with osteoporosis treated with antiresorptive drugs requiring dental care. In recognizing the close relationship between osteoporosis treatment and advanced dental care, a task force with representation from the *Sociedade Brasileira de Endocrinologia e Metabologia* (SBEM; Brazilian Society of Endocrinology and Metabolism), *Sociedade Brasileira de Estomatologia e Patologia Oral* (Sobep; Brazilian Society of Stomatology and Oral Pathology), and *Associação Brasileira de Avaliação Óssea e Osteometabolismo* (Abrasso; Brazilian Association for Bone Evaluation and Osteometabolism) convened to perform a comprehensive review of available evidence and publish a joint consensus document on the issue providing up-to-date recommendations for clinical practice.

Osteoporosis is a highly prevalent disease resulting in fragility fractures that reduce quality of life and increase the risk of death. This condition affects mainly postmenopausal women but may also affect older men. Osteoporosis may be secondary to several chronic diseases, including inflammatory disorders (rheumatic, intestinal, and respiratory), malignant conditions (multiple myeloma), endocrine diseases (hypogonadism, primary hyperparathyroidism, and Cushing's syndrome), and conditions that cause intestinal malabsorption ( 3 , 5 ). Medications can also cause osteoporosis, including glucocorticoids, which are the most common cause of secondary osteoporosis ( 3 ). In Brazil, available data confirm that osteoporosis incurs in significant individual and social burden: approximately 15% of female and 13% of male Brazilian adults present fragility fractures ( 6 ), and the annual costs for treatment of osteoporotic fractures may amount to 310 million US dollars ( 7 ).

The American Association of Oral and Maxillofacial Surgeons (AAOMS) defines MRONJ as “The presence of exposed jaw bone, or bone that can be probed through an intraoral or extraoral fistula, for at least 8 weeks, in a patient with a history of antiresorptive and/or antiangiogenic therapy, and in the absence of previous radiation therapy to the head and neck” ( 8 ). Complications that might be expected in patients with MRONJ are local infection, pain, loss of function, and poor quality of life ( 3 ).

Although rare and more commonly seen in patients with cancer, MRONJ is associated with substantial morbidity that can cause anguish to patients and health care professionals, making therapeutic decisions difficult. Therefore, identifying patients at risk of MRONJ and the factors that may predict the development of this complication is extremely important. Here, we discuss the risk of MRONJ and preventive strategies in patients with osteoporosis receiving antiresorptive therapy and undergoing invasive dental procedures.

## MATERIALS AND METHODS

An electronic search of the literature was performed to identify published studies that reported MRONJ and other adverse effects of antiresorptive therapy in patients with osteoporosis from January 2002 to September 2019. Clinical and epidemiological studies reporting on the risk and prevention of MRONJ in patients with osteoporosis in the English language were eligible for the analysis. Reviews, letters to the editors, studies that did not disclose the type of antiresorptive therapy, animal studies, and those not available in the full version were not included.

A multidisciplinary task force was convened including representatives of three Brazilian professional associations with interest in bone metabolism and oral care: the *Sociedade Brasileira de Endocrinologia e Metabologia* (SBEM; Brazilian Society of Endocrinology and Metabolism), *Sociedade Brasileira de Estomatologia e Patologia Oral* (Sobep; Brazilian Society of Stomatology and Oral Pathology), and *Associação Brasileira de Avaliação Óssea e Osteometabolismo* (Abrasso; Brazilian Association for Bone Evaluation and Osteometabolism). Specific questions were proposed and addressed by members of the task force related to the literature review. A draft position was reviewed by coordinators of the three societies, and the task force unanimously approved the final report.

### Osteoporosis treatment and adverse effects

Approved therapeutic approaches to prevent and treat osteoporosis modify bone remodeling by either slowing the rate of bone loss (antiresorptive) or increasing the rate of bone formation ( 9 ). A variety of antiresorptive agents are available, acting through different mechanisms to reduce bone loss. Therefore, the therapeutic choice is guided by pharmacologic characteristics, desired antiresorptive properties, potential benefits, adverse effects, and context-dependent availability. Based on the aim of this paper, only bisphosphonates and denosumab will be discussed, as these antiresorptive agents have been previously associated with MRONJ.

Bisphosphonates are the most common medications used for osteoporosis treatment worldwide, and alendronate, risedronate, ibandronate, and zoledronic acid are the four available molecules approved for this purpose. Bisphosphonates have a very high affinity for the bone mineral matrix, where they deposit and are taken up by osteoclasts during active bone resorption ( 5 ). The osteoclast cytoskeleton is disarranged by bisphosphonates, leading to cell apoptosis and inhibition of bone resorption and remodeling.

Besides MRONJ, other adverse effects have been associated to bisphosphonates. Esophageal and gastric dyspeptic symptoms are the most common adverse effects of these agents, but atypical femoral fractures and atrial fibrillation have also been reported ( 10 , 11 ). Since the skeleton can retain bisphosphonates for up to 10 years, depending on the bone turnover rate, the effects of these agents last for months or even years after discontinuation of these drugs ( 12 ). This long half-life allows for a more convenient therapeutic dosage, including weekly, monthly, quarterly, or annual regimens, along with periodic drug holidays in low-risk patients ( 5 ).

Denosumab is a human monoclonal antibody directed against the receptor activator of nuclear factor-κB ligand (RANKL), thereby inhibiting the differentiation and activity of osteoclasts and greatly suppressing bone resorption ( 13 ). Denosumab is administered as a single subcutaneous injection every 6 months. Unlike bisphosphonates, denosumab does not accumulate in the skeleton and its effects cease immediately upon treatment discontinuation ( 13 ). A rebound effect may be observed, with an increased risk of fragility fractures within 12 months from the suspension of this drug ( 14 ).

### MRONJ epidemiology in patients with osteoporosis

The prevalence of MRONJ in patients receiving oral bisphosphonates for the treatment of osteoporosis ranges from 0-0.04%, with most reports citing prevalences below 0.001% ( 15 ). A total of 1.04-69 cases of MRONJ per 100,000 patients treated with oral bisphosphonates are described yearly. In clinical trials, the risk for MRONJ among patients treated with either zoledronic acid (0.017%) or denosumab (0.04%) is comparable to the risk observed in patients assigned to placebo (0-0.02%). Thus, based on the current literature, the risk of developing MRONJ among patients with osteoporosis exposed to oral or intravenous bisphosphonates or denosumab is real but remains very low and almost the same or slightly higher than the risk (0.001%) in the general population ( 16 ). The duration of antiresorptive therapy has also been demonstrated as a risk factor for the development of MRONJ ( 8 ). Even though the causes of secondary osteoporosis, such as the use of glucocorticoids, can pose additional risks for MRONJ, this complication can occur in both primary and secondary osteoporosis ( 17 ).

A recent Brazilian cross-sectional study evaluated 153 patients taking oral or intravenous bisphosphonate for osteoporosis treatment and 134 individuals using bisphosphonates for metastatic breast cancer. A 3% prevalence of MRONJ in women with metastatic breast cancer receiving bisphosphonates was identified, while no case of MRONJ was observed in patients receiving these agents for osteoporosis ( 18 ).

Reliable epidemiological evidence on the incidence of MRONJ is particularly challenging to obtain due to several limitations regarding sample sizing (generally small), study design (retrospective rather than prospective), study duration, and lack of active case-finding strategies. Underreporting of MRONJ may also lead to inaccurate incidence rates.

### Influence of systemic factors on MRONJ development

Several clinical factors other than antiresorptive therapy have been associated with MRONJ, including diabetes mellitus, rheumatoid arthritis, hypertension, smoking, and use of other medications such as glucocorticoids, antithrombotic agents, immunosuppressants, and proton pump inhibitors ( 19 , 20 ) ( Table 1 ).

**Table 1:** Main risk factors for medication-related osteonecrosis of the jaw

Systemic risk factors	
Diabetes mellitus	
Rheumatoid arthritis	
Hypertension	
Smoking	
Medications:	
	Antiresorptive agents
	Antiangiogenic agents
	Glucocorticoids
	Antithrombotic agents
	Immunosuppressants
	Proton pump inhibitors
**Local risk factors**	
Dental infection	
Trauma	
Invasive oral procedures	

Long-term use of bisphosphonates and better adherence to treatment may also increase the risk of MRONJ ( 15 , 16 ). A Korean study of patients with osteoporosis reported a significant association between the use of bisphosphonates and the risk of MRONJ, with a dose-response relationship and a higher risk among patients exposed to bisphosphonate therapy for 1.5-2 years compared with those exposed for 0-1.5 year ( 21 ). A longitudinal cohort of 61,990 users of alendronate followed up for a mean period of 6.8 years identified 107 cases of MRONJ and showed that recent compared with past use of alendronate was associated with an adjusted odds ratio of 4.13 (95% confidence interval [CI] 1.94-8.79) ( 20 ). The risk of MRONJ was two to three times increased in adherent users with a medication possession ratio greater than 50%. Finally, patients on long-term use of alendronate (>5 years) showed a higher risk of MRONJ than those on short-term use of this drug ( 20 ).

### Influence of local factors in the development of MRONJ

The maxillary bones are highly susceptible to MRONJ since bisphosphonates accumulate mainly in skeletal sites with high bone remodeling activity and because the thin oral mucosa can be easily traumatized ( 17 , 18 ). MRONJ affects the mandible more frequently than the maxilla and is more common in some areas, such as bony prominences, tori, and mylohyoid line ( 22 ).

Inflammatory oral disease, such as active periodontal conditions, caries, acute or chronic pulpoperiapical processes, trauma from removable prosthesis or other physical trauma, and poor oral hygiene, are considered local risk factors for MRONJ ( 23 ). The mouth is colonized by a large number of bacteria, and the jaw bones are often involved in infectious processes of periodontal or pulp origin. When bisphosphonates accumulate in these sites, tissue repair following induced or physiological trauma fails to occur properly, potentially leading to an area of exposed necrotic bone in the oral environment ( 23 ).

All patients starting antiresorptive therapy should be referred to dentists for preventive oral procedures and orientation prior to initiating the osteoporosis treatment. Once patients are receiving antiresorptive therapy, oral surgical interventions, especially dental extractions, are considered predisposing factors associated with MRONJ. A growing body of evidence suggests that dental infection, rather than dental extraction per se, might represent the main local risk factor for MRONJ ( 24 ). Considering tooth extraction, those performed traumatically or without primary closure of the socket are more related to the development of MRONJ ( 25 , 26 ).

### Drug holiday for MRONJ prevention

Based on the residual effects of bisphosphonates after long-term therapy, patients at low fracture risk after 5 years of alendronate or 3 years of zoledronic acid can undergo a drug holiday with periodic reassessment. This recommendation aims to minimize safety concerns ( 27 ). However, a retrospective population study in a Korean database showed a higher prevalence of MRONJ in the first 3 years after bisphosphonate suspension ( 28 ). Although patients with and without cancer were studied, no correlation between dosing frequency or time since bisphosphonate discontinuation and the occurrence of MRONJ was observed in either group ( 28 ).

The risk of developing MRONJ is small compared with the antifracture benefits of bisphosphonates in individuals with moderate to high fracture risk using these drugs ( 29 ). So far, no evidence-based study has demonstrated efficacy of bisphosphonate drug holiday in preventing MRONJ.

Denosumab has no sustained antiresorptive effect. Hence, drug holiday is not recommended with this medication, mainly due to recent concerns about a rapid increase in the risk of vertebral fractures following denosumab discontinuation ( 30 ).

Only one study has aimed to evaluate the effect of interrupting oral bisphosphonates before invasive dental procedures for the prevention or treatment of MRONJ ( 31 ). In this study, 1,175 patients underwent 2,458 surgical procedures. Some patients underwent the procedure after 2 or 3 months of bisphosphonate withdrawal, and no significant difference in the frequency of MRONJ occurred between patients who did or did not discontinue the medication before tooth extraction.

There is no evidence supporting the suspension of antiresorptive therapy for osteoporosis before dental procedures for preventing MRONJ ( 31 ). Position papers from several professional societies and task forces do not include a recommendation for routine discontinuation of bisphosphonate therapy for osteoporosis before invasive dental procedures ( 15 , 16 , 24 , 27 ). Additionally, a multicenter retrospective study has shown that the suspension of bisphosphonates has no benefit on the outcome of patients with osteoporosis who developed MRONJ ( 32 ).

### Predictive factors for MRONJ

Several studies have attempted to assess the risk of MRONJ using bone turnover markers (BTMs). Marx and cols. ( 33 ), in a retrospective, uncontrolled study of 30 patients, have proposed that C-terminal telopeptide (CTX) levels measured before dental surgery could predict MRONJ. Since then, many other studies have been carried out to investigate the ability of BTMs to predict the occurrence of MRONJ, with conflicting results ( 34 – 36 ).

A recent systematic review and meta-analysis of 18 clinical trials involving 2,301 patients receiving bisphosphonates and scheduled for dental surgery showed no significant difference in mean CTX levels between patients who developed MRONJ and those who did not develop this complication ( 37 ). Also, no significant differences occurred in the prevalence of MRONJ between patients with CTX levels lower or higher than 150 pg/mL (p = 0.92). Based on these data, the authors concluded that the measurement of CTX levels for identifying patients at risk of MRONJ is not justified by evidence, and the proposed CTX cutoff value of 150 pg/mL is not associated with an increased prevalence of this complication ( 37 ). Therefore, current evidence does not support using CTX levels before dental procedures for predicting the risk of MRONJ.

Other serum biomarkers were recently assessed in a systematic review ( 38 ) analyzing six biomarkers: the bone turnover biomarkers bone alkaline phosphatase, deoxypyridinoline, N-telopeptides of type I collagen, and osteocalcin; the endocrine biomarker parathyroid hormone; and the angiogenesis marker vascular endothelial growth factor. All biomarkers analyzed failed to predict the risk of MRONJ adequately.

Quantitative trabecular and cortical bone measurements have shown to be potentially useful tools in detecting bone changes caused by bisphosphonates in mixed populations of patients taking oral or intravenous bisphosphonate ( 39 , 40 ). In the studies, which evaluated only patients taking oral bisphosphonates for osteoporosis, radiographic and cone-beam computed tomography measurements were not predictive of MRONJ ( 41 , 42 ).

Qualitative evaluation of the jaw bones, such as mandibular cortical index evaluated on panoramic radiographs, has also been shown to be useful in predicting the development of MRONJ in patients with osteoporosis ( 43 ). Sclerosis of trabecular bone and thickening of the lamina dura were considered imaging features consistent with bisphosphonate administration ( 43 ).

### Tooth extractions in patients with osteoporosis using antiresorptive medication

Performing a dental extraction procedure on a patient using an antiresorptive agent requires care before, during, and after the procedure. The preoperative procedures involve preparing the oral environment, with supragingival scaling, removal of active carious lesions, and orientation of oral hygiene, whenever indicated ( 44 ).

Antibiotic prophylaxis should be initiated prior to tooth extraction, and amoxicillin with or without clavulanate is most commonly indicated for this purpose ( 45 ). For patients allergic to penicillin, clindamycin may be prescribed. A systematic review has reported different prophylactic regimens starting 48 to 72 hours before the procedure and maintained for 1 to 3 weeks after the procedure ( 45 ). There is no uniform approach applied to all patients receiving bisphosphonates with regard to the length of prophylactic antibiotic treatment before and after tooth extraction, but most authors agree that post-extraction treatment regimens should be continued until the surgical site has completely healed ( 45 ). Antibiotic therapy should be maintained for a longer period if surgical wound repair is delayed ( 23 ).

The surgical technique to be performed should be minimally invasive and should provide primary closure of the dental socket ( 23 ). Alveoloplasty may facilitate primary closure of the socket after extraction, but surgeons have full discretion in deciding whether to perform it or not ( 23 ). The development of MRONJ seems to be related to cases in which the dental alveolus is not primarily closed in the immediate postoperative period, therefore requiring more healing time ( 23 ). Careful postoperative follow-up is required until the healing process is complete, which may take longer than usual ( 46 ).

**Figure 1:**
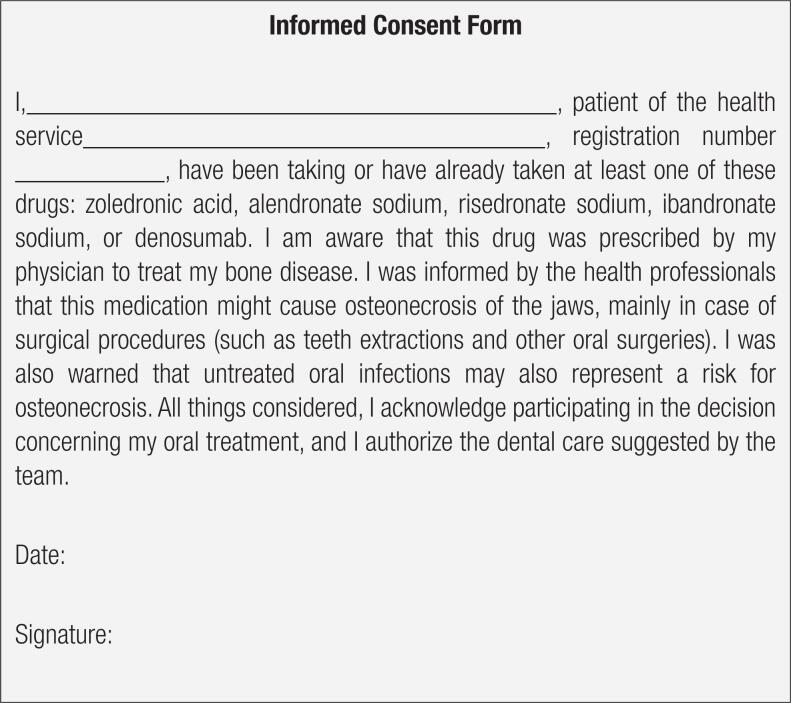
Informed consent form model for patients undergoing osteoporosis treatment who will undergo invasive dental treatment.

Another important recommendation is the completion of an informed consent form, which should make the patient aware of the low risk of dental extraction for the development of MRONJ, and the importance of complying with the recommendations on the postoperative follow-up ( 47 ). Figure 1 shows an example of an informed consent form for patients receiving osteoporosis treatment who will undergo invasive dental treatment.

### Implant placement in patients with osteoporosis undergoing antiresorptive therapy

The risk of MRONJ after dental implant placement is considered to be comparable to the risk of MRONJ associated with tooth extraction ( 8 ). Based on current recommendations, the placement of dental implants should be avoided in patients receiving intravenous antiresorptive or antiangiogenic therapy, whereas treatment with oral antiresorptive drugs is not considered an explicit contraindication for the procedure ( 48 ). However, caution is advised in view of conflicting data regarding implant failure and the risk for MRONJ development in patients with osteoporosis.

A study with 235 women with osteoporosis receiving oral bisphosphonates who underwent dental implant placement and concomitant application of plasma rich in growth factor reported no cases of MRONJ and a survival implant rate of 98.7% ( 49 ). Successful dental implant installation has also been reported in patients using alendronate for 3 years who received antibiotic prophylaxis for 7 days before the procedure, with favorable osseointegration observed in 98% of the patients ( 50 ).

Despite the low risk of MRONJ with oral bisphosphonates, only a few studies have accurately defined the risk of developing MRONJ in the presence of dental implants. Approximately 20% and 80% of the patients who developed MRONJ in the vicinity of dental implants have osteoporosis and oncologic disease, respectively ( 51 ). Although the risk of MRONJ is significantly higher in patients using intravenous bisphosphonates, an association between peri-implantitis and the etiology of MRONJ can also be observed in patients who use oral bisphosphonates ( 48 , 51 ).

The use of minimally invasive techniques and soft tissue bone coverage have been described as factors that considerably reduce the incidence of complications after dental implant treatment ( 50 ). Measures such as avoiding bone augmentations and administration of antimicrobial prophylaxis are also recommended in these patients ( 52 ).

Since infection around the implants represents a notable risk factor for MRONJ development, the implant presence itself – and not only the implant surgery – may trigger MRONJ ( 3 , 43 , 46 ). In the informed consent form, patients should confirm that they are aware of possible long-term implant failure and the risk of MRONJ on implant sites ( Figure 1 ) ( 8 , 47 ).

### Endodontic, periodontal, and orthodontic treatment in patients with osteoporosis receiving antiresorptive therapy

A controlled clinical trial on the risk of MRONJ related to conservative root canal treatment showed satisfactory outcomes such as periradicular healing in patients taking long-term oral bisphosphonates ( 53 ).

Although periodontal disease may benefit from systemic bisphosphonate therapy ( 54 ), the disease itself is considered a risk factor for MRONJ ( 55 ). Anaerobic bacteria representative of periodontal microbiota were the main findings in the necrotic bone samples collected from MRONJ cases, suggesting that periodontal infection could actually initiate the process in patients undergoing antiresorptive treatment ( 56 ).

No studies have attributed orthodontic treatment to increased risk of MRONJ. While no clinical studies have been designed to specifically address orthodontic treatment in patients taking antiresorptive drugs for osteoporosis, one report has described three patients using oral bisphosphonates who, during orthodontic treatment, presented decreased tooth movement and difficulty for space closure and paralleling of roots ( 57 ).

Biologically, bisphosphonates can impact orthodontic treatment by impeding tooth movement due to osteoclast destruction and decreased microcirculation, limiting bone turnover and remodeling ( 58 ). Patients receiving bisphosphonate therapy must be aware of difficulties in closing the extraction spaces and paralleling the roots ( Figure 1 ) ( 47 ).

### MRONJ management in patients with osteoporosis

The AAOMS position paper recommends nonsurgical local treatment as the therapy of choice for early-stage MRONJ ( 8 ). However, surgical treatment is recommended for lesions refractory to initial treatment and those at an advanced stage. Bone sequestrations that are a constant source of soft tissue irritation and loose bony sequestra should be removed or recontoured so that soft tissue healing can be optimized ( 8 ).

In a study using the AAOMS protocol to treat patients with MRONJ, cure was achieved in 93% of the cases ( 59 ). However, only 34% of the sample showed improvement with nonsurgical treatment alone. Surgical procedures were performed in 62% of the patients and were effective in 94% of the sequestrectomies and 100% of the resections. In another study, conservative or surgical treatment was effective in 72% of the patients ( 60 ). Conservative treatment was refractory in 44% of the lesions, while surgical treatment was effective in all cases.

Surgical procedure can be used as the first line of treatment or in lesions refractory to conservative treatment ( 59 , 61 ). However, surgeries under intravenous sedation or general anesthesia have shown better results, and patients with MRONJ could benefit from treatments more invasive than bone curettage performed under local anesthesia ( 61 ). The surgical treatment associated with the use of platelet-rich fibrin has also been effective, but it is not possible to ascertain if the cure was caused by the surgical procedure or by adjuvant biological materials ( 62 ). Recent studies have proposed surgeries to be performed even in the early stages of the disease, with a better prognosis ( 24 ).

Reports related to the medical treatment of MRONJ available in the literature are mostly case reports or small case series. The results of these studies are generally favorable to the use of teriparatide, although no definite answer for this approach is available due to the small number of patients treated and the lack of a control group in most reports ( 63 , 64 ). These studies show improvement in serum markers or bone regeneration ratio, but do not show resolution of MRONJ.

In contrast to these previous series, a study that compared the local application of recombinant human bone morphogenetic protein-2 (rhBMP-2) alone or combined with short-term teriparatide versus no treatment (control group) observed that patients who received rhBMP-2 plus teriparatide had better outcomes than those who received rhBMP-2 alone or controls, suggesting that the administration of rhBMP-2 may maximize bone regeneration after surgery ( 63 ). More studies are still needed to recommend pharmacological treatment for the management of MRONJ.

In conclusion, antiresorptive medications are the most used pharmacological treatment for osteoporosis and reduce the risk of all types of fragility fractures. Although only scarce evidence links the development of MRONJ to the use of antiresorptive agents in the context of osteoporosis, these agents are considered a risk factor for MRONJ. Despite rare reports of MRONJ in patients with osteoporosis taking antiresorptive drugs, the severity of the impact of MRONJ in patients' quality of life makes it imperative for appropriate diagnosis, prevention, and treatment of this complication. Health care professionals should always work together to improve patients' safety and attain better outcomes.
